# Computed Tomography Evaluation of Coronary Atherosclerosis: The Road Travelled, and What Lies Ahead

**DOI:** 10.3390/diagnostics14182096

**Published:** 2024-09-23

**Authors:** Chadi Ayoub, Isabel G. Scalia, Nandan S. Anavekar, Reza Arsanjani, Clinton E. Jokerst, Benjamin J. W. Chow, Leonard Kritharides

**Affiliations:** 1 Department of Cardiovascular Medicine, Mayo Clinic, Phoenix, AZ 85054, USA; 2 Department of Cardiovascular Medicine, Mayo Clinic, Rochester, MN 55905, USA; 3 Department of Radiology, Mayo Clinic, Phoenix, AZ 58054, USA; 4 Department of Medicine (Cardiology), University of Ottawa Heart Institute, Ottawa, ON K1Y 4W7, Canada; 5 Department of Radiology, University of Ottawa, Ottawa, ON K1Y 4W7, Canada; 6 Department of Cardiology, Concord Hospital, Sydney Local Health District, Concord, NSW 2137, Australia

**Keywords:** coronary artery disease, coronary computed tomography angiography, coronary artery calcium scoring, artificial intelligence

## Abstract

Coronary CT angiography (CCTA) is now endorsed by all major cardiology guidelines for the investigation of chest pain and assessment for coronary artery disease (CAD) in appropriately selected patients. CAD is a leading cause of morbidity and mortality. There is extensive literature to support CCTA diagnostic and prognostic value both for stable and acute symptoms. It enables rapid and cost-effective rule-out of CAD, and permits quantification and characterization of coronary plaque and associated significance. In this comprehensive review, we detail the road traveled as CCTA evolved to include quantitative assessment of plaque stenosis and extent, characterization of plaque characteristics including high-risk features, functional assessment including fractional flow reserve-CT (FFR-CT), and CT perfusion techniques. The state of current guideline recommendations and clinical applications are reviewed, as well as future directions in the rapidly advancing field of CT technology, including photon counting and applications of artificial intelligence (AI).

## 1. Introduction

Imaging is essential for the accurate evaluation of cardiovascular diseases and to guide further patient management. Over recent decades, cardiac computed tomography (CT) has had increasing clinical utility and widespread use in this setting, propelled by rapid advances in technology and techniques. Coronary CT angiography (CCTA) is the most widely used application of this modality. There is a plethora of literature to support its diagnostic accuracy, its and prognostic value, its ability to improve health outcomes compared to usual care, and its role in resource allocation by facilitating more appropriate use of preventive medications and triaging invasive coronary angiography [1,2]. In clinical practice, CCTA has proven to be safe, improves diagnostic yield and certainty, and enhances risk stratification, particularly in subjects with non-obstructive coronary artery disease [2,3,4,5,6,7]. 

CCTA has evolved such that it enables quantitative assessment of plaque stenosis and extent and allows for characterization of plaque characteristics, including high-risk features. Its utility for functional assessment is now well established, with fractional flow reserve-CT (FFR-CT) and CT perfusion techniques. CT technology continues to advance, allowing improved characterization of disease with new technologies such as photon counting CT detectors and applications of artificial intelligence (AI). This review will cover the capabilities of CCTA, including its evolution, role and clinical utility, prognostic value in patient care, and future directions.

## 2. Coronary Atherosclerosis

Coronary artery disease (CAD) is the primary cause of death globally [8,9]. The morbidity associated with CAD is also significant on an individual patient level in terms of symptoms and clinical events, and as well as by way of economic burden that is borne by the community [10]. As such, imaging techniques that may detect, diagnose, and risk-stratify CAD are important for management and to help attenuate this burden.

In the clinical setting, risk factors such as hypertension, dyslipidemia, diabetes, smoking history and family history of premature coronary artery disease, or combined risk scores such as the Framingham risk score or the pooled cohort risk equation for Atherosclerosis Cardiovascular Disease (ASCVD), have been used to predict risk for CAD [11]. However, clinical risk factors may not always identify at-risk patients, and clinical risk scores, including the ASCVD score, may overestimate risk. Consequently, imaging modalities are utilized to improve the identification of CAD and to incrementally enhance risk stratification.

## 3. CCTA in the Context of Imaging Evaluation for CAD

Currently available investigations for CAD are broadly divided into functional and anatomic tests, as summarized in Table 1. Stress electrocardiography and echocardiography, single-photon emission computed tomography (SPECT), positron emission tomography (PET), and stress cardiac magnetic resonance (CMR) imaging are considered ‘functional’ non-invasive tests to evaluate for ischemia [12]. In addition, PET and CMR may provide information regarding myocardial viability to help guide revascularization decisions [13,14]. Invasive coronary angiography (ICA) is the gold standard anatomic diagnostic test for CAD evaluation, with invasive FFR and instantaneous flow reserve (iFR) considered the gold standard for evaluation of the functional significance of stenosis [15,16].

CCTA is predominantly an anatomic, non-invasive imaging test that, similar to ICA, can directly visualize the coronary arteries and describe both the location and category of stenosis [19,20,21]. Functional evaluation can also be acquired from CCTA from techniques such as FFR-CT. Clinically, CCTA’s strength lies in its very high sensitivity, yielding a high negative predictive value for CAD [17,22]. Multiple trials and meta-analyses have demonstrated the diagnostic accuracy of CCTA, studied in symptomatic populations with predominantly low or intermediate risk (Supplementary Table S1). The EVINCI and PICTURE multicenter prospective studies, conducted in European and United States centers, respectively, showed that CCTA had excellent diagnostic accuracy for detecting significant coronary stenosis when compared with functional imaging modalities (with ICA as the gold standard) [1,23].

Depending on the population being examined, elective ICA may have a large number of normal tests or tests showing hemodynamically minor coronary disease that does not require coronary intervention [24]. As such, CCTA has an increasing role as ‘gatekeeper’ to the catheterization laboratory, with its excellent capability to accurately exclude the presence of significant coronary disease and minimize the number normal ICAs in appropriately selected patients. Clinically, CCTA has since evolved to play an important role not only in the detection of CAD, but also in its quantification and risk stratification. This is of particular relevance in patients with non-obstructive CAD, whereby the clinical value of CCTA allows not only for avoidance of unnecessary invasive coronary catheterization, but also promotes the opportunity for better risk factor control, particularly with statin therapy, which may reduce mortality [5,6].

### 3.1. Reductions in Radiation Exposure

Improvements in CT technology, including increasing detector rows, prospective electrocardiogram (ECG) gating for image acquisition, more powerful X-ray tubes enabling scanning at lower kVp, advances in CT image reconstruction such as deep learning iterative reconstruction, and a better understanding of best scanning practice including heart rate control, have combined to result in a drastic reduction in effective radiation exposure [25]. On current scanning systems employing best practice recommendations, CCTA would be expected to result in an effective radiation range of 1–5 mSv [26]. This compares favorably with an average of about 1 mSV for the coronary calcium score [27], 5–10 mSv for stress and rest Tc-99 SPECT, 2–3 mSv for stress and rest PET perfusion, and a mean of 7 mSv (can range from 2–23 mSv depending on case complexity) for ICA [28].

### 3.2. Transition from Qualitative to Quantitative CCTA Evaluation

Clinical reporting of CCTA has previously focused on qualitative evaluation of CAD. There was a focus on articulating if disease was present or absent, and if present, classifying plaque as either obstructive or non-obstructive, and plaque composition as calcified, non-calcified or mixed. However, in 2016 the Society of Cardiovascular Computed Tomography (SCCT) introduced the first CAD-RADS (Coronary Artery Disease Reporting and Data System) guideline, endorsing a quantitative reporting of CCTA to standardize communication findings and therefore improve clinical decision making [19]. These recommendations articulate numeric grading of severity of plaque stenosis, and description of plaque features, including high-risk plaque features, with standardized management recommendations (Table 2).

More recently, the SCCT has released an updated guideline: CAD-RADS 2.0 (Table 2) [29]. Following widespread uptake and validation of the CAD-RADS guideline, which has allowed not only for standardized reporting of CAD but also prognostic utility and reduction in invasive intervention of non-obstructive CAD, the CAD-RADS 2.0 guideline has been updated to include changes such as integration of plaque burden scores and modification of diagnostic variables such as high-risk plaque features or evidence of ischemia on perfusion imaging [30].

### 3.3. Plaque Characteristics

Historically, CAD risk has been defined only by degree of stenosis on ICA. However high-risk features previously identified by intravascular ultrasound (IVUS) and on autopsy studies have since been evaluated and characterized on CCTA [17]. Specifically, CCTA has been validated in assessing coronary atherosclerotic plaque characteristics that may suggest plaque vulnerability and predict acute coronary syndromes (ACS) or clinical events, independently of the degree of stenosis [31,32,33].

**Figure 1:**
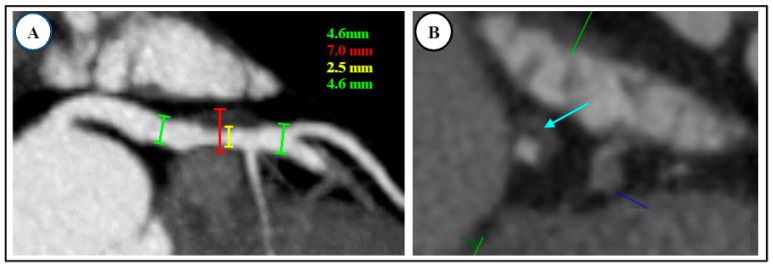
High-risk plaque features on coronary CT angiography (CAD RADS = 3 + HRP). HRP = High-risk plaque. (**A**) Positive remodeling seen in proximal left anterior descending artery (red bracket), with moderate (50–60%) underlying stenosis (2.5 mm luminal diameter shown with the yellow line). Outer caliber of artery is >1.5 times the inner lumen; 7.0 mm total vessel diameter is shown with the red line, and the green lines with 4.6 mm measurements demonstrate the luminal diameter pre- and post-stenosis. (**B**) Low-attenuation lipid core (<30 Hounsfield units, light blue arrow) with speckled calcium.

Evaluation of coronary artery stenosis severity by CCTA can be both qualitative (mild, moderate, severe) and quantitative (measures include % diameter stenosis, % area stenosis, minimal luminal diameter, and minimal luminal area). High-risk plaque characteristics include low attenuation plaque (tissue attenuation ≤30 Hounsfield Units [HU]), spotty calcification (speckles of calcium ≤3 mm), plaque volume, plaque diffuseness, positive remodeling (diseased/normal outer diameter ≥1.1), and plaque composition (non-calcified > calcified), demonstrated in Figure 1A [31,33,34,35,36,37]. In addition, the napkin ring sign, defined as a low attenuation core (<30 HU) surrounded by a rim of higher attenuation (but less than <130 HU), has been described as a high-risk feature (Figure 1B) [38]. The presence of more than one high-risk feature confers increased risk for ACS [32,36,39].

High-risk plaque features have better potential to predict events at four-year follow-up than the presence of ≥70% stenosis or prior ACS, and the presence of high-risk plaque features and stenosis together confers even higher risk [32]. In addition, the study by Motoyama et al. demonstrated that plaque progression on serial CCTA (defined as increase in stenosis by ≥1 grade or increase in the remodeling index ratio of >1.1) signified the greatest risk of all for myocardial infarction [32].

Atherosclerotic plaque features have also been shown to help predict the presence of ischemia. Positive remodeling has been associated with ischemia in plaques both with <50% and >50% stenosis, and plaque volume and low attenuation are more common in ischemic plaques with >50% stenosis [40]. For lesions of intermediate severity, plaque volume has additionally been shown to be incremental in predicting ischemia [41], and ‘plaque diffuseness’ (length of plaques in a coronary artery) may show vessel-specific ischemia independent of CAD severity and other high-risk plaque characteristics [42].

More recently, the perivascular fat attenuation index (FAI), a marker on CCTA that may indicate coronary artery inflammation, has been investigated. It has been observed that inflammation shifts composition of perivascular coronary tissue from the lipid (more negative HU, around −190 HU), to the liquid phase (less negative, around −30 HU) [43]. The CRISP CT study identified a cut point of −70 HU of FAI in the right coronary artery perivascular territory to be incrementally prognostic to state-of-the-art CCTA assessment, with an HR of 5.2 (95% CI 2.90–10.88, *p* < 0.0001) for cardiac mortality in the validation cohort of the study [43]. Further studies are required to further understand the clinical role of this novel high-risk imaging marker.

Given the validation of these high-risk features, the initial CAD-RADS reporting guideline included the modifier “V” to represent vulnerable or high-risk plaque if ≥2 such features are observed. The specific high-risk features that CAD-RADS includes are low attenuation plaque, positive remodeling, spotty calcification, and the “napkin ring sign” [19]. Subsequently, the recent CAD-RADS 2.0 reporting guidelines adjusted this modifier to “HRP” for high-risk plaque features. Another significant update in these new guidelines was the introduction of plaque burden grading, which was not present in the initial CAD-RADS [29]. Overall, the summative quantity of plaque has significant prognostic implications, irrespective of plaque characteristics or severity of stenosis [44,45]. Quantification of plaque burden may be conducted with multiple methods, including coronary artery calcium (CAC) score, visual estimation, or segment involvement score (SIS) [29].

## 4. Functional Evaluation by CT

Despite the strengths of CCTA, it can have limitations in the clinical setting. There is a tendency to overestimate stenosis severity; in particular, heavily calcified lesions may be challenging to quantify [46]. Accordingly, there have been advances in the functional component to CT evaluation in tandem with its anatomic assessment. Functional CAD evaluation by CT primarily includes FFR-CT, and some centers may also utilize CT perfusion (CTP) imaging [47]. The transluminal attenuation gradient has also been used to grade coronary lesion hemodynamic significance by measuring the decrement in HU attenuation across lesions [48].

Given the increasingly promising data for both CTP and CT-FFR, these modalities have been included in the updated CAD-RADS 2.0 reporting guideline for the assessment of ischemia and related reversibility, added to the score as “I” [29]. In conjunction with the other components of the CAD-RADS 2.0 score, inclusion of ischemia may then guide further invasive investigation and management.

### 4.1. Fractional Flow Reserve—Computed Tomography (FFR-CT)

Invasive FFR measurement, using pressure wires across a stenotic lesion on ICA, is the gold standard for evaluating hemodynamic significance [15]. Non-invasively, FFR-CT is a technique that applies computational fluid dynamics to estimate the hemodynamic significance of a stenosis seen on CCTA, with values <0.8 signifying ischemia [47]. Typically, anatomic CCTA images of the coronary arteries are sent to an offsite company that analyzes them with this technique and returns FFR-CT results to the referring center. No additional protocols or medications are required; however, the anatomic imaging needs to be of a reasonable quality to apply FFR-CT.

**Figure 2:**
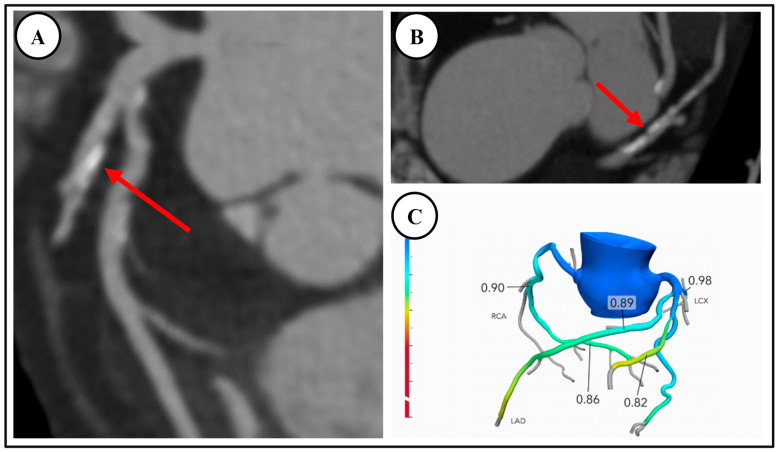
A 75-year-old outpatient with stable angina, with CT demonstrating moderate CAD without ischemia. (**A**,**B**) Coronary CT angiography demonstrated moderate coronary artery disease, stenosis 50% with mixed disease in the proximal and mid-left anterior descending artery, red arrows (CAD-RADS = 3). (**C**) FFR-CT negative for obstructive disease. Patient subsequently underwent stress echocardiography which was negative for inducible ischemia. He was commenced on aggressive risk factor modification.

**Figure 3:**
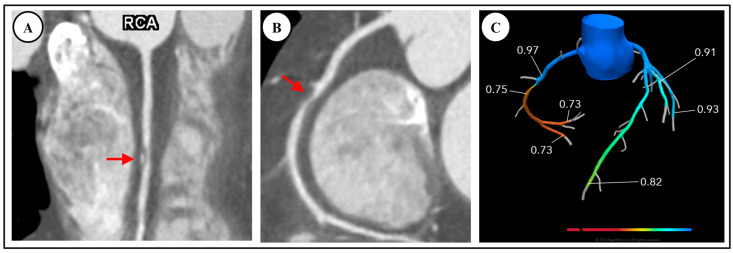
A 59-year-old male reviewed in the outpatient clinic with stable angina and multiple cardiovascular risk factors, found to have moderate CAD with ischemia. (**A**,**B**) Linear and curved multiplanar reconstructions on CCTA respectively demonstrate moderate stenosis in right coronary artery (RCA), red arrows, reported as 50–70% (CAD-RADS = 3). (**C**) FFR-CT positive for ischemia and suggesting obstructive coronary artery disease in right coronary artery. Patient was referred for invasive coronary angiography.

There are robust data supporting the diagnostic accuracy of FFR-CT to determine lesion-specific hemodynamic significance. Clinically, this is demonstrated in Figure 2, whereby a case of moderate CAD on CCTA was confirmed on FFR-CT as non-obstructive. Conversely, Figure 3 demonstrates a similar case of moderate CAD on CCTA, found to be obstructive on subsequent FFR-CT. The DeFacto multicenter study compared FFR-CT to invasive FFR in 252 stable, predominately symptomatic patients with suspected or known CAD, and found that FFR-CT enhanced diagnostic accuracy to 73% compared to 64% with obstructive CAD seen on CCTA alone, whereas FFR-CT plus CCTA had a sensitivity of 90% and specificity of 54% [49]. The NXT and Discover Flow studies also showed that FFR-CT improved the detection of lesions causing ischemia compared to CCTA alone (with invasive FFR as the reference standard) [50,51]. A meta-analysis found that FFR-CT had an overall diagnostic accuracy of 82% but suggested that accuracy varied considerably depending on the range of FFR-CT values. There was less diagnostic accuracy for FFR-CT values between 0.7–0.8, and increasing accuracy for values both above or below this range [52].

Another meta-analysis, comparing all anatomic and functional tests for CAD with invasive FFR as the reference, found that although CCTA had high sensitivity, its anatomic evaluation was limited in specificity for ischemia. FFR-CT increased specificity from 0.4 for anatomic CCTA to predict hemodynamically significant lesions to 0.7, which is comparable to SPECT and stress echocardiography with specificities of around 0.8 (however, the latter do not offer anatomic information) [13]. The same meta-analysis, however, found that (stress) MRI had the best diagnostic performance for identifying ischemia causing coronary lesions [13].

Several studies have further evaluated the clinical utility and impact of FFR-CT, demonstrating improved efficiency in triaging patients to ICA compared to CCTA alone. The Platform study demonstrated that FFR-CT when combined with CCTA is safe, with no events in the 61% of CCTA/FFR-CT patients in whom ICA was cancelled [53]. It also demonstrated that FFR-CT was associated with significantly less ‘no obstructive CAD’ on ICA (12% for CCTA plus FFR-CT compared to 73% in the usual care/ICA arm) [53], and was accompanied by lower resource utilization and cost at 90 days [54].

The Ripcord and PROMISE studies have shown that the addition of FFR-CT reclassified management decisions in about a third of patients presenting with stable chest pain, and improved prediction for revascularization and clinical events compared to CCTA findings alone [55,56]. Registry data also confirm that positive FFR-CT in general correlates with the presence of severe stenosis; however, it importantly showed that it reclassified some severe stenoses as being non-ischemic, and some ‘mild’ stenoses by CCTA luminal evaluation as being functionally significant [57].

Although FFR-CT holds much potential, further studies are needed to better understand its role in patients with known CAD (including stents and coronary artery bypass grafts). The main limitation of the current commercially available FFR-CT is the need for offsite analysis. It is likely that in the future, CT systems will incorporate an inbuilt, less technically demanding version of FFR-CT for on-site application [58]. FFR-CT may facilitate a future in which the catheterization laboratory becomes a predominately therapeutic center for lesions which are hemodynamically predefined by CCTA. Presently, there are efforts underway to incorporate AI to predict FFR values from CCTA images.

### 4.2. CT Perfusion Imaging (CTP)

CTP has emerged as another functional component of the CT evaluation for CAD. Akin to nuclear myocardial perfusion techniques, CTP evaluates the left ventricle for areas of ischemia at rest and post stress with a chemical agent such as regadenoson or adenosine, which are reflected by areas of contrast hypo-attenuation or perfusion defects. CTP techniques include static, single image acquisition during myocardial perfusion, or dynamic, multiple image acquisition throughout the cardiac cycle and semi-quantitative evaluation of myocardial blood flow (MBF) [59,60,61]. Technically, some advantages of stress CTP imaging include simultaneous anatomic and perfusion data and high spatial resolution with data acquired for the entire ventricle.

Diagnostically, CTP has been validated for evaluation of both acute and stable chest pain, compared with other functional investigations including SPECT and MRI perfusion, as well as ICA and FFR [29]. Overall, the diagnostic sensitivity has been suggested to be up to 0.89 and specificity ranging from 0.78 to 0.95; dynamic stress CTP when compared with static stress CTP had higher pooled specificity (0.89 vs. 0.78), but lower pooled sensitivity (0.77 vs. 0.82) [62,63,64,65,66,67,68]. Furthermore, combined assessment with CCTA and single-phase stress acquisition CTP had improved diagnostic performance [68,69,70].

The diagnostic utility of CTP has further been demonstrated in multiple prospective studies, in comparison to ICA and SPECT imaging, particularly in the setting of triple vessel disease and left main disease [61,71,72]. Additionally, data from CRESCENT-II suggested that a staged CAC score, which if positive followed by CCTA, which in turn is followed by CTP if a ≥50% coronary stenosis is present, was associated with better triage to ICA and revascularization than standard functional testing with stress echocardiography [73]. Prognostically, several studies have demonstrated the utility of CTP alone or in combination with CCTA in predicting cardiovascular adverse events, similar to that of ICA and SPECT [74].

Despite the diagnostic and prognostic value of CTP, some disadvantages include potential artifacts (motion and beam hardening), greater contrast load, need for the administration of a vasodilator, more complex preparation protocols, greater preparation and scan time, as well as higher ionizing radiation dose [70]. As such, currently, CTP is not widely used as FFR-CT; however, it presents a reasonable alternative imaging option. Additionally, cardiac PET and stress MRI are still considered the most accurate modalities for quantitative perfusion assessment [75].

## 5. Subclinical CAD by CCTA

Although obstructive CAD confers the poorest prognosis for cardiac events and death, patients with non-obstructive coronary atherosclerosis in turn have a poorer outcomes compared to those with no CAD, as identified by either CCTA or ICA [76]. Significantly, a greater extent of non-obstructive atherosclerosis or a greater number of involved coronary vessels on CCTA has also been associated with increasing adverse events [77,78,79,80]. The ability of CCTA to directly visualize non-obstructive coronary lesions is a strength when compared to functional imaging modalities, which are unable to detect these, as coronary lesions need to be flow-limiting and obstructive in order to be detected by way of regional wall motion abnormality (RWMA) with stress echocardiography or perfusion defect in nuclear imaging.

The importance of identifying non-obstructive CAD by CCTA lies in the opportunity afforded to intervene early in the disease process to help reduce disease progression and clinical events. Given that the extent of non-obstructive CAD on CCTA is related to the risk of cardiac events, even in the absence of any obstructive lesions [45], CCTA further risk-stratifies patients for optimal preventative therapy. In clinical practice, CCTA has been shown to improve aspirin and statin use for risk prevention, and increasing severity of CAD by CCTA has been linked with improved adherence to medical treatment and superior risk factor control [2,81]. There is retrospective data from large cohorts to suggest that treatment with statin may reduce the risk of clinical events in those with non-obstructive CAD seen by CCTA [5,6]. For this reason, CAD-RADS 2.0 includes the Segment Involvement Score (SIS), which gives a sense of disease burden of CAD even in the absence of significant stenosis [29,82].

### Coronary Artery Calcium Score

The CAC score has been used widely as a surrogate for CAD burden and as a robust tool for predicting risk. It involves a low-dose gated-CT acquisition that requires lower radiation exposure, and no contrast administration compared to CCTA, but is not able to define if obstructive stenosis is present. Overall, CAC score testing has been shown to be both effective and cost-saving as a risk-stratification tool, enabling more precise targeting of preventative therapies based on the patient’s risk of CAD [83]. There is an extensive volume of literature confirming the prognostic value of the CAC score by the Agatston method. The landmark Multi-Ethnic Study of Atherosclerosis (MESA) study showed that CAC can stratify cardiovascular events in diverse populations [84]. Increasing CAC score is associated with progressively higher event rates, and long-term data have validated its prognostic power [27].

There is substantial evidence that the CAC score improves risk stratification over clinical risk evaluation and the Framingham score [85], and can help stratify whether to treat people more or less aggressively [86,87]. The ASCVD pooled cohort equation, which replaced the Framingham score, may be imprecise in certain groups and may, in some cases, overestimate risk [88]. A CAC score of zero confers excellent prognosis, and strongly portends low likelihood of development of ASCVD [89]. Guidelines have recommended statin therapy if the 10-year ASCVD risk is >7.5%; however, incorporation of CAC in these intermediate-risk patients may allow for reclassification and, as such, CAC is included in current guideline recommendations [11]. Specifically, the absence of CAC reclassifies nearly half of patients falling in this category as not requiring statin therapy, which has important appropriate use and economic implications [90]. The CAC score has been shown to improve risk factor control, decrease downstream testing, and enhance adherence to preventative measures [91,92].

The utility of CAC scoring is particularly relevant in asymptomatic patients with cardiovascular risk factors. In these cases, the CAC score may reclassify patients as high- or low-risk, and therefore alter the approach to preventative therapy (Table 3) [11,93]. SCCT guidelines and the 2019 American Heart Association (AHA) Guidelines for the Primary Prevention of Cardiovascular Diseases endorse the use of the CAC score in shared decision making in asymptomatic, intermediate-risk groups (“asymptomatic individuals without clinical ASCVD who are 40–75 years of age with 10-year ASCVD risk between 5–20%”), as well as “selectively in the <5% ASCVD group [low risk], such as in those with a family history of premature coronary artery disease” [11,94].

Furthermore, recent evidence has supported the use of CAC scoring in low-risk patients presenting with acute chest pain. In 2022, Grandhi et al. described a negative predictive value of 99.3% for the diagnosis of obstructive CAD in patients with a CAC score of zero, who presented with acute chest pain and were deemed low- or intermediate-risk for CAD [98]. This has further been reported in a subsequent meta-analysis, with the negative predictive value of a CAC score of zero to be 97% for CAD in low- or intermediate-risk patients with stable chest pain and 98% in patients with acute chest pain [99]. As such, the most recent AHA/American College of Cardiology (ACC) chest pain guideline has included the CAC score (without contrast enhanced CCTA) for the first time as a suitable test to rule out CAD in acute chest pain in low-risk patients [26]. Irrespective, clinical judgment on a case-by-case basis is still recommended in the acute chest pain setting, as a positive CAC score in a low-risk patient with chest pain will require further testing to exclude ischemia. Additionally, younger patients (particularly under the age of 40) may have soft plaque, which may be missed by the CAC score alone.

## 6. Clinical Role of CCTA

The high sensitivity and negative predictive value of CCTA for CAD enhances its clinical utility (Supplementary Table S1) [1,23,100,101,102,103,104,105,106]. Numerous studies and meta-analyses have confirmed excellent prognostic value for the absence of CAD by CCTA, and better prognosis in subjects with non-obstructive CAD compared to those with obstructive CAD [107,108,109,110]. Improvements in diagnostic accuracy and firm data for prognostic value have led to contemporary widespread clinical use of CCTA, which includes evaluation for the presence, extent, and severity of stenosis, and plaque composition [29,79,108,109].

CCTA has established roles in the Emergency Room (ER) for evaluation of acute chest pain, in the outpatient setting for chronic stable chest pain, and also in the inpatient setting to evaluate for CAD in a number of different scenarios. Current guideline indications for CCTA include evaluation of patients with chest pain or ischemic equivalent symptoms that have low-to-intermediate cardiovascular risk, or asymptomatic patients only if there is a family history of premature coronary disease [20,26,111]. CCTA is not, however, currently recommended in asymptomatic individuals without significant family history, based on data from the CONFIRM registry showing no incremental risk stratification value compared to the CAC score alone [112,113]. The SCOT-HEART 2 trial is currently underway to prospectively evaluate whether a screening CCTA in the asymptomatic population would have clinical value [114].

**Figure 4:**
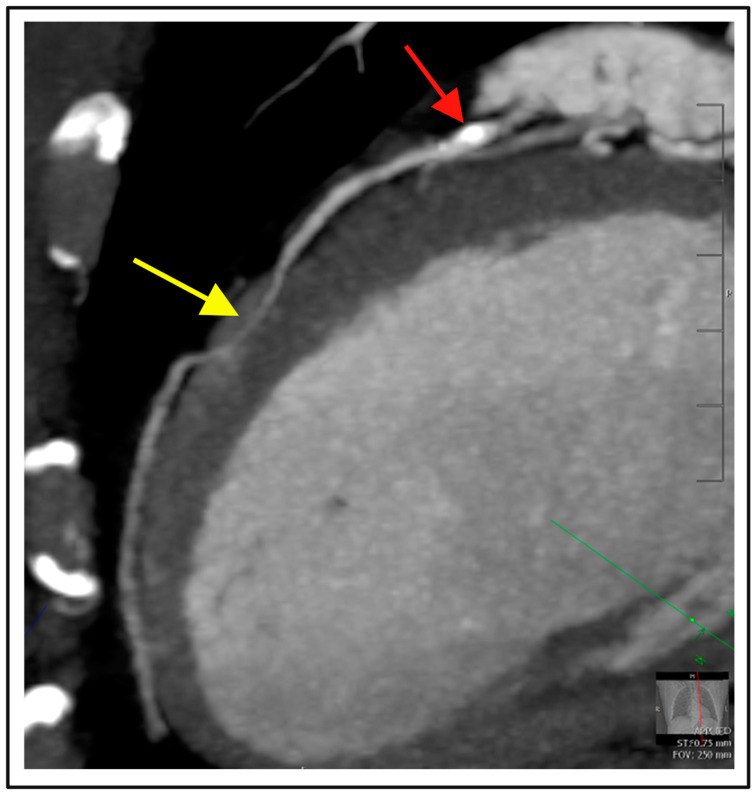
Nonobstructive coronary artery disease. A 67-year-old male with a history of hyperlipidemia presented with subacute atypical chest pain. CCTA excluded obstructive coronary artery disease. Curved multiplanar reconstruction demonstrates mild stenosis in the proximal left anterior descending artery (25–50% stenosis, red arrow) with myocardial bridging in the mid segment (yellow arrow). Patient was accordingly commenced on statin therapy.

There are a number of other increasingly used indications for CCTA in both the inpatient and outpatient settings [115], including the exclusion of CAD as the cause of heart failure in patients being investigated for impaired systolic function. CCTA may also allow for exclusion of obstructive coronary disease in scenarios where the likelihood for CAD is low, for example, with suspected myocarditis, stress cardiomyopathy, myocardial bridging, or in coronary spasm, as demonstrated in Figure 4 [14,26,116]. Anatomic visualization to assess for suspected anomalous coronary anatomy is a strength of CCTA, and where present, high-risk features of anomalous coronary artery such as an ‘interatrial course’ or “slit like ostial opening” can be further assessed.

In the preoperative setting for cardiac valvular surgery, when evaluating whether concomitant bypass grafting may be required, CCTA can be used to exclude CAD in low-risk patients [14,117]. For example, CCTA is useful prior to aortic valve replacement for bicuspid aortic valve disease, where patients tend to be younger and atherosclerotic coronary disease is less likely [118]. CCTA is also excellent in the evaluation of patency of coronary grafts [118]. CCTA is generally not recommended for coronary stent evaluation due to artifacts from the stent beams, particularly those with stented segments of <3 mm diameter [115].

CCTA is most suitable in patients with no known prior history of CAD, or in those with recurrent symptoms who have had negative prior functional tests. CCTA would not be appropriate in patients with suspected acute MI or in patients with high clinical risk, for whom ICA would be appropriate. Symptomatic patients with known CAD would be more suitably investigated with either functional testing or ICA, depending on the clinical context, and CCTA may also not be helpful or diagnostic in those with known high CAC score or diffuse coronary calcification. Older patients or those with advanced renal disease would likely fall into this category.

### 6.1. CCTA in the Emergency Room

**Figure 5:**
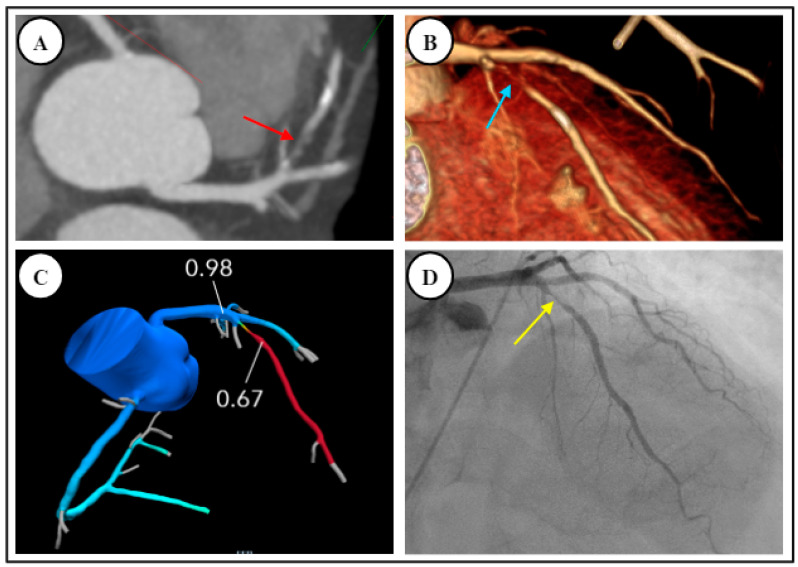
Obstructive coronary artery disease. A 79-year-old male presented to Emergency Room with two-week history of chest pain. (**A**,**B**) CCTA demonstrated severe stenosis with mixed plaque in left anterior descending artery (CAD-RADS = 4) on axial imaging, red arrow, and 3D reconstruction, light blue arrow, respectively. (**C**) FFR-CT positive for ischemia with a value of 0.67. (**D**) Invasive coronary angiography confirmed severe obstructive disease, yellow arrow; patient proceeded to percutaneous coronary intervention.

CCTA is increasingly utilized in hospital ERs both because of its ability to be performed rapidly and its high negative predictive power. Clinically, event rates in symptomatic patients approach zero if CCTA rules out significant coronary stenosis [26,119,120]. Patients with CCTA-guided treatment of acute chest pain, including referral to ICA, have also been shown to have fewer events at 18 month follow-up when compared to patients undergoing functional testing [121]. An example of this is seen in Figure 5.

Five major CCTA trials in the ER (CT-STAT, ACRIN, ROMICAT II, BEACON and the Study of Coronary Artery Computed Tomography to Diagnose Emergency Chest Pain) and meta-analyses of four of these have shown that CCTA significantly shortens the length of the ER stay, reduces the time to diagnosis, and reduces costs compared to the usual diagnostic approach (without CCTA) including ICA [3,4,22,122,123,124]. Both meta-analyses reported no increase in the rate of ICA, but did reveal an increased likelihood for revascularization, suggesting that CCTA may better select patients for the catheterization laboratory [3,4]. Overall, these studies have comprehensively demonstrated the value of CCTA to rule out CAD and prevent invasive investigation in low-to-intermediate-risk patients presenting with acute chest pain.

The 2015 European Society of Cardiology (ESC) Non-ST-Elevation Myocardial Infarction Guidelines initially introduced CCTA as an alternative to ICA in the ER setting, with IIa (level A) recommendation for rule-out of ACS when there is a low-to-intermediate likelihood of CAD, and the troponin test and electrocardiogram (ECG) are inconclusive [125]. Subsequently, the 2021 AHA Guideline for the Evaluation and Diagnosis of Chest Pain have recommended CCTA as the first-line investigation of acute chest pain in intermediate-risk patients with no known history of CAD with inconclusive evidence of ACS (Class I evidence, level A), or in intermediate-risk patients with a previous history of mildly abnormal functional CAD testing or non-obstructive CAD (class IIa) [26]. The updated CAD-RADS 2.0 Guideline, as well as the 2023 ESC Guidelines for the management of acute coronary syndromes, has also been adapted for the incorporation of CCTA in acute chest pain work-up [29,126].

**Figure 6:**
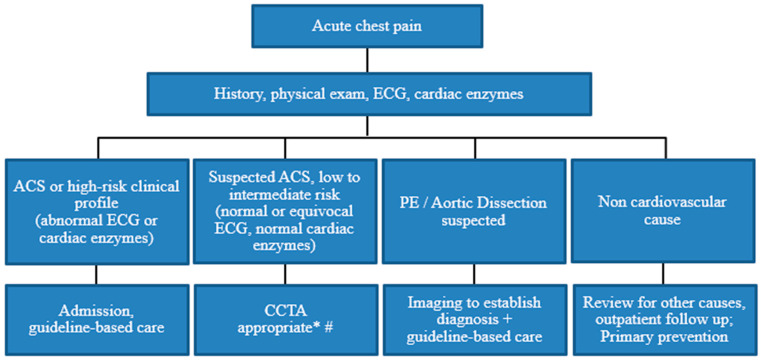
Suggested algorithm for the approach to acute chest pain in the Emergency Room. * If no contraindication. # Can consider triple rule-out if PE or aortic dissection is suspected. Abbreviations: ACS = acute coronary syndrome, CCTA = cardiac computed tomography angiography, ECG = electrocardiogram, PE = pulmonary embolism.

Another clinical role for CCTA in the ER, after exclusion of high-risk ACS, is to evaluate chest pain with suspected cardiac etiology without coronary atherosclerotic pathology based on the clinical presentation (for example, stress cardiomyopathy, myocarditis, or coronary spasm), whereby CCTA can effectively and rapidly exclude obstructive coronary stenosis. A suggested algorithm for the potential role of CCTA for the evaluation of acute chest pain in the ER is demonstrated in Figure 6, based on current recommendations [12,26,126].

### 6.2. CCTA in the Outpatient Setting for Stable Chest Pain, Compared to Functional Testing

There is well-established independent and incremental diagnostic and prognostic value of CCTA in patients with stable chest pain and suspected CAD in the outpatient setting [79,127]. The exclusion of obstructive CAD with CCTA identifies patients with lower event rates, and the absence of CAD is associated with excellent intermediate-term prognosis. Overall, this enables CCTA to act as a gatekeeper, reducing unnecessary ICA and procedure-related complications in low-to-intermediate-risk patients with stable chest pain [16,128,129,130,131,132]. These findings have been replicated in the large, multicenter, multinational CONFIRM registry, which enrolled 27,125 patients undergoing CCTA [110,133].

Multiple large randomized multicenter prospective trials have sought to better understand the clinical usefulness of CCTA in outpatients with stable chest pain (Supplementary Table S2) [7,21,134,135]. In particular, the SCOT-HEART and PROMISE trials were practical real-world trials that evaluated the impact of anatomic testing by CCTA compared with stress testing in all-comers with chest pain and suspected CAD [7,21]. PROMISE randomized 10,000 subjects to CT versus physician-selected stress testing (mostly nuclear), with scans locally read, whilst SCOT-HEART compared standard-of-care, including exercise testing, compared with CCTA in over 4000 subjects.

In both trials, the use of CCTA was associated with similar hard events as the more established functional testing in landmark analyses for follow-up time of about two years [7,21]. Although not its primary endpoint, SCOT-HEART almost reached statistical significance in showing that CCTA was accompanied by a reduction in fatal and non-fatal MI by 38% after 1.7 years follow-up, *p* = 0.0527 [7]. However, subsequent analyses from the same data have suggested that CCTA may be superior in predicting hard event outcomes. The PROMISE trial, whose primary composite endpoint was all-cause mortality, MI, hospitalization for unstable angina and procedural complications, when stratified by presence of obstructive versus non obstructive CAD, showed that CCTA and functional testing had similar prognostic value for those with obstructive CAD, but CCTA provided better prognostic information than functional testing by identifying patients at risk because of non-obstructive CAD [136].

SCOT-HEART showed that CCTA use was associated with decreased cardiovascular death and MI, after a median lag time of about 50 days to initiation of preventative therapy was removed (Hazard ratio [HR]: 0.50, *p* = 0.020) [2]. On longer-term five-year follow-up data, CCTA in addition to standard care afforded a lower rate of death from CAD or nonfatal MI in those with stable chest pain compared to standard care alone (2.3% vs. 3.9%; hazard ratio 0.59, *p* = 0.004), without any associated significant increase in coronary catheterization or revascularization [80].

SCOT-HEART also showed that CCTA increased diagnostic certainty, its primary outcome [7]. Although neither trial required or monitored optimal medical therapy nor guideline-directed care, both showed that CCTA improved clinical decision-making. This was by way of improved patient selection for invasive procedures and higher catheterization yield. CCTA was shown to better guide use of preventative therapy but did not improve lifestyle changes in these studies. A post hoc analysis of SCOT-HEART showed four-fold increase in the appropriate use of aspirin and/or statin in those who had CCTA that showed disease, as well as cancellation of therapy when CCTA was normal [2].

SCOT-HEART had a similar rate of patients who underwent ICA in both groups, which differed from PROMISE, which had higher ICA rate in the CCTA arm. SCOT-HEART observed a 60% lower rate of normal coronary arteries on catheterization in the CCTA group, and those who had CCTA were 30% more likely to have obstructive CAD, which is similar to PROMISE, where the CCTA arm had a higher rate of revascularization [2]. The higher rate of revascularization in the CCTA group may be a marker of better selection and more appropriate use of catheterization; however, appropriate use was not directly evaluated.

Data from these trials are consistent with existing large cohort data that suggests that CCTA is an effective gatekeeper to the catheterization laboratory. CONFIRM demonstrated that high-risk CAD found on CCTA that is subsequently revascularized is associated with survival benefit [133,137]. The National Cardiovascular Data Registry (NCDR) data reviewing 661,063 patients showed that CCTA was associated with lower rates of non-obstructive CAD findings and the higher rates of obstructive CAD on ICA when compared to ECG exercise stress test (EST), stress echocardiography, and nuclear functional imaging [138].

Other recent trials have also compared CCTA with exercise treadmill testing, including CRESCENT with 350 patients and CAPP with 500 patients followed for about a year [134,135]. Both studies, like PROMISE and SCOT-HEART, showed higher diagnostic yield for CCTA, and CCTA was associated with less angina (likely explained by consequent increased use of preventative and anti-ischemic therapies), earlier time to diagnosis, less downstream testing and cost, but no difference in hard events [134,135].

Consequent to these large studies, recent guidelines, including the 2024 ESC Guidelines for the management of chronic coronary syndromes, incorporate CCTA for diagnosis of obstructive CAD in patients with low-moderate pre-test probability (>5–50%) of obstructive CAD [139]. It is further recommended with Class IA evidence for the estimation of risk of major adverse cardiac events in these patients.

### 6.3. Socio-Economic Impact of CCTA on Clinical Management

In the current environment of value-based health care, CCTA appears to have similar overall cost-effectiveness compared to traditional care and functional testing, but confers value with enhanced clinical decision making, efficiency and prognostic information. PROMISE showed that CCTA had similar costs to functional testing through three years of follow-up [140]. SCOT-HEART showed a higher cost early with CCTA; however, there was no significant difference in long-term or downstream costs in the context of better-guided care [2]. CRESCENT and CAPP reported a cost saving with CCTA, and this was also reported in the CONSERVE trial, likely a result of avoided ICAs [135,141,142].

In the acute care setting, some studies have previously associated the use of CCTA with higher catheterization and revascularization rates [143]. The ROMICAT II trial observed higher downstream testing with use of CCTA in the ER, but also noted improved efficiency in clinical decision making with no difference in cumulative cost compared to standard care [124]. A subsequent meta-analysis has shown that CCTA use was accompanied by decreased ER cost and length of stay, but, however, also reported increased ICA and revascularization [3].

## 7. Future Directions

### 7.1. Photon Counting CT (PCCT) Technology

PCCT scanners are the next frontier in the long history of CT technological advancement. This technology will serve as an avenue for significant improvements in diagnostic accuracy due to the increased spatial resolution, lower level of background noise, and early evidence for more accurate plaque burden quantification compared to CAC scoring [144]. Furthermore, in addition to high spatial resolution capabilities of PCCT detectors (down to 0.2 mm), PCCT is capable of spectral imaging, allowing for material decomposition reconstructions. This additional capability may allow for detailed evaluation of the myocardium, including quantification of perfusion defects [145,146]. With such diagnostic utility, PCCT has the potential to significantly change the landscape of CAD evaluation, allowing for earlier and more efficient risk stratification, reducing the number of patients requiring invasive investigation, and providing an accurate and reproducible gate-keeper for the ongoing management of patients with CAD [144,147].

### 7.2. Artificial Intelligence Applications in CCTA and CAC

In the era of automated data capture and AI, applications including radiomics and deep learning have the potential to transform clinical CCTA and CAC, not only in regard to improved reproducibility, image optimization, and speed of interpretation, but also in enhancing prognostic utility [148,149,150].

Multiple facets of CCTA evaluation of CAD present opportunities for AI input and advancements. Firstly, multiple studies have validated the accuracy of AI, specifically convolutional neural networks (CNN) and artificial neural networks (ANN), to localize and grade the severity of coronary artery stenosis on CCTA, alone or in hybrid with perfusion imaging, in comparison with invasive FFR [149,151]. The accuracy for detection of stenosis in these models ranged from 0.67–0.94 [152,153]. Furthermore, AI models have been assessed in the evaluation of plaque phenotype [154]. As well as differentiating between calcified or non-calcified plaque, ANN- and radiomic-based models have been described to identify high-risk plaque features such as napkin-ring sign, with accuracy of ranging from 0.77 to 0.89 [152,155].

Assessment of ischemia via CT perfusion and CT-FFR have also been evaluated using AI models, in comparison to invasive assessment [154]. Machine learning (ML) models applied to CT-FFR studies have been demonstrated to have not only a high diagnostic accuracy, 0.84–0.89, but were also noted to significantly reduce computational time compared to traditional fluid dynamic methods [156,157,158]. However, as with traditional FFR-CT assessment, results were found to be significantly dependent on CT image quality [149].

AI models have also been established and validated for the quantification of plaque burden [151]. These models have been found to decrease processing time when compared to traditional methods, with good accuracy for detection of a CAC score ≥100 on both gated and non-gated CT chest imaging [159,160]. Furthermore, deep learning (DL) algorithms have been applied to non-gated CT chest images, for example, PET-CT or radiation therapy planning CT chest images, with, again, very good accuracy (intraclass correlation coefficient up to 0.97 compared to manual CAC scoring) [161]. Clinically, these represent valuable real-world opportunities for opportunistic identification of CAD, as well as improved diagnostic accuracy on a range of different scan images.

As a summation of these AI models, several groups are developing DL algorithms directly for CAD-RADS reporting [162]. Initial results found an accuracy of 0.86 in the detection of CAD-RADS 0 versus CAD-RADS >0, with accuracy slightly lower at 0.71 in distinguishing CAD-RADS 0–2 from CAD-RADS 3–5. The analysis time for the CT studies was significantly faster with the application of this AI model [163]. Further, Johnson et al. demonstrated the prognostic utility of AI to be superior to CAD-RADS in 6892 patients for all-cause mortality (AUC 0.77 vs. 0.72), death related to CAD (AUC 0.85 vs. 0.79), and cardiac death or myocardial infarction (AUC 0.85 vs. 0.80) [164]. To date, AI models for evaluation of CAD-RADS have utilized only the initial CAD-RADS reporting guidelines, with updated algorithms for CAD-RADS 2.0 not yet reported on.

Additionally, ML-AI models have demonstrated incremental prognostic utility compared to CCTA. Motwani et al. showed the predictive ability of AI in the large CONFIRM population for CAD, with AUC accuracy of 0.79 compared to traditional scoring metrics such as the clinical Framingham Risk Score with AUC of 0.61, or the segment stenosis score (SIS) with AUC of 0.64 [165]. Furthermore, other aspects of CCTA have become feasible targets for AI modelling. Radiomics and AI models have been evaluated for the detection of subendocardial scarring, as well as quantification of epicardial/perivascular fat, both of which have been previously shown to be prognostic markers [154,166].

Although these AI advancements hold much promise, many obstacles yet need to be overcome, including quality and representative datasets to train AI platforms and identifying areas where AI technology may be more prone to errors or inaccuracies. Whilst AI holds the potential to streamline workflow, reduce costs and radiation exposure, and enhance the diagnostic and predictive power of many imaging modalities, it will not obviate the need for expert human clinicians to over-read and confirm interpretation of the imaging.

## 8. Summary of Clinical Practice

Patients with low-to-intermediate risk with chest pain or equivalent symptoms are the optimal candidates for CCTA. If CCTA demonstrates no CAD or non-obstructive coronary atherosclerosis, then the need for ICA is alleviated. Additionally, in those with non-obstructive CAD, preventative measures including statin therapy can be initiated, which can improve prognosis. In those with obstructive CAD or with moderate lesions with positive FFR-CT, CCTA can better triage to proceed to ICA for further evaluation and potential intervention.

## 9. Conclusions

CCTA is in widespread clinical use as an accurate non-invasive imaging modality for the detection and evaluation of CAD and is included in guideline recommendations for this purpose in appropriately selected patients. Although it has great utility with is its high negative predictive value and ability for effective rule-out of CAD, it has evolved into an effective tool for quantification of CAD, plaque characterization, and prognostication. The ability to characterize high-risk plaque features has further enhanced the clinical value of CCTA, and functional information with FFR-CT and CTP helps better define the hemodynamic significance of lesions identified.

Accordingly, high-risk plaque features, the presence of ischemia, and semi-quantitative scores that give indication of plaque stent (including nonobstructive plaque) have accordingly been incorporated in the recent updated CAD-RADS 2.0 clinical reporting recommendations. Advances in CT technology such as the introduction of high-resolution PCCT offers increased accuracy and spatial resolution for the evaluation and quantification of CAD. Applications of AI are anticipated to improved reproducibility, image optimization, and speed of interpretation.

## Figures and Tables

**Table 1:** Investigative modalities for coronary artery disease [12,17,18].

	Advantages	Limitations
**Functional**		
**Stress ECG**	Non-invasive, no radiation, readily available, low cost	Poor localization, requires exercise capacity to achieve adequate stress
**Stress TTE**	No radiation, high availability, localizes ischemia, concurrent assessment of other parameters such as valvular function or diastology	Body habitus, requires technical expertise, requires adequate exercise capacity or tolerance of dobutamine
**SPECT (stress/rest imaging)**	High availability, localizes ischemia, highly reproducible, cost effective	Attenuation artifacts in obesity, false positives, balanced ischemia, exposure to radiation
**PET (stress/rest imaging)**	High accuracy (particularly in patients with high BMI), can assess myocardial blood flow	Limited availability, expense, exposure to radiation
**Stress MRI perfusion**	High accuracy, no ionizing radiation exposure, assessment of myocardial scar	Low availability, time consuming, device incompatibilities, expense, contraindicated in severe renal dysfunction
**Anatomical**		
**Invasive coronary** **angiography**	High accuracy, widely available, allows for contemporaneous intervention	Invasive, requires intravenous contrast
**Coronary Computed** **Tomography Angiography**	High accuracy, rapid assessment, identification of subclinical disease	Arrhythmias, high heart rates requiring beta-blocker therapy, stents, renal impairment, severe coronary calcification may cause blooming artifact, exposure to radiation, contraindicated in severe renal dysfunction
**MRI angiography**	High spatial resolution, no ionizing radiation, highly reproducible	Low availability, time consuming, device incompatibilities, contraindicated in severe renal dysfunction

Abbreviations: ECG = electrocardiogram, MRI = magnetic resonance imaging, PET = positron emission tomography, SPECT = single positron emission computed tomography, TTE = transthoracic echocardiography.

**Table 2:** CAD-RADS 2.0 guideline summary for stable coronary artery disease (reproduced with permission) [29].

	Degree of Maximal Coronary Artery Stenosis	Interpretation	Further Investigation	Further Management Considerations (Incorporates Plaque Burden Score from CAC Scoring, Refer to Table 3)
**CAD-RADS 0**	0%	CAD absent (no plaque)	None	Reassurance
**CAD-RADS 1**	1–24%	Minimal non-obstructive plaque	None	P1: Consider risk factor modification and preventative therapy P2: Risk factor modification and preventative therapy P3: Aggressive risk factor modification and preventative therapy
**CAD-RADS 2**	25–49%	Mild non-obstructive CAD	None	P1/2: Risk factor modification and preventative therapy P3/4: Aggressive risk factor modification and preventative therapy
**CAD-RADS 3**	50–69%	Moderate stenosis	Consider functional assessment	P1–4: Aggressive risk factor modification and preventative therapy Consider anti-anginal therapy Consider ICA if positive perfusion testing (I+)
**CAD-RADS 4**	A. 70–99% B. LM ≥ 50% or three vessel disease ≥ 70%	Severe stenosis	A: Consider ICA or functional test B: ICA recommended	P1–4: Aggressive risk factor modification and preventative therapy Consider anti-anginal therapy +/− revascularization
**CAD-RADS 5**	100%	Total coronary occlusion	Consider ICA and/or viability assessment	P1–4: Aggressive risk factor modification and preventative therapy Consider anti-anginal therapy +/− revascularization
**CAD-RADS N**	Non-diagnostic study	Study cannot exclude significant obstructive CAD		

Reproduced with permission from the Society of Cardiovascular Computed Tomography 2022 Coronary Artery Disease Reporting and Data System 2.0 Guidelines [29]. CAD-RADS modifiers: N = Non-diagnostic. Abbreviations: CAC = coronary artery calcium, CAD = coronary artery disease, ICA = invasive coronary angiography, LM = Left Main coronary artery.

**Table 3:** Treatment considerations based on CAC score, incorporating CAD-RADS 2.0 updated classification of plaque burden [11,29,93,95,96,97].

CAC Score	CAD-RADS 2.0 Classification of Plaque Burden	Statin Recommendation and Intensity	Aspirin for Primary Prevention	Blood Pressure Targets in Intermediate-Risk Patients *
**0**		Statin likely of limited value (except for those with suspected familial hypercholesteremia), assess other risk factors	Harm likely > benefits, not recommended	Goal BP < 130/80 mmHg
**1–99**	P1 Mild overall coronary plaque burden	**CAC < 75th%**: Moderate-intensity statin **CAC ≥ 75th%**: Moderate- to high-intensity statin	Considering clinical risk, unlikely to benefit from aspirin	Goal BP < 130/80 mmHg
**100–299**	P2 Moderate overall coronary plaque burden	Moderate to high intensity	Aspirin recommended following assessment of bleeding risk	Goal BP < 130/80 mmHg
**300–999**	P3 Severe overall coronary plaque burden	High-intensity statin	Aspirin recommended following assessment of bleeding risk	Goal BP < 130/80 mmHg
**>1000**	P4 Extensive overall coronary plaque burden			

* ASCVD risk ≥ 10%. Abbreviations: ASCVD = atherosclerotic cardiovascular disease, BP = blood pressure, CAC = coronary artery calcium, CAD = coronary artery calcium, RADS = Reporting and Data System.

## Supplementary Materials

The following supporting information can be downloaded at: https://www.mdpi.com/article/10.3390/diagnostics14182096/s1, Table S1: Diagnostic accuracy for stenosis by coronary computed tomography angiography. Table S2: Major trials evaluating clinical impact of coronary computed tomography angiography in patients with stable chest pain.
